# Treatment and outcome of Shiga-toxin-associated hemolytic uremic syndrome (HUS)

**DOI:** 10.1007/s00467-008-0935-6

**Published:** 2008-10-01

**Authors:** Johanna Scheiring, Sharon P. Andreoli, Lothar Bernd Zimmerhackl

**Affiliations:** 1 Department of Pediatrics I, Medical University Innsbruck, Anichstr. 35, A-6020 Innsbruck, Austria; 2 James Whitcomb Riley Hospital for Children, Indianapolis, IN 46202 USA

**Keywords:** Enterohemorrhagic *Escherichia coli* (EHEC), Hemolytic uremic syndrome, Diarrhea, Shiga toxin (Stx)1 and Stx2, Complement, Complications

## Abstract

Hemolytic uremic syndrome (HUS) is the most common cause of acute renal failure in childhood and the reason for chronic renal replacement therapy. It leads to significant morbidity and mortality during the acute phase. In addition to acute morbidity and mortality, long-term renal and extrarenal complications can occur in a substantial number of children years after the acute episode of HUS. The most common infectious agents causing HUS are enterohemorrhagic *Escherichia coli* (EHEC)-producing Shiga toxin (and belonging to the serotype O157:H7) and several non-O157:H7 serotypes. D^+^ HUS is an acute disease characterized by prodromal diarrhea followed by acute renal failure. The classic clinical features of HUS include the triad of microangiopathic hemolytic anemia, thrombocytopenia, and acute renal failure. HUS mortality is reported to be between 3% and 5%, and death due to HUS is nearly always associated with severe extrarenal disease, including severe central nervous system (CNS) involvement. Approximately two thirds of children with HUS require dialysis therapy, and about one third have milder renal involvement without the need for dialysis therapy. General management of acute renal failure includes appropriate fluid and electrolyte management, antihypertensive therapy if necessary, and initiation of renal replacement therapy when appropriate. The prognosis of HUS depends on several contributing factors. In general “classic” HUS, induced by EHEC, has an overall better outcome. Totally different is the prognosis in patients with atypical and particularly recurrent HUS. However, patients with severe disease should be screened for genetic disorders of the complement system or other underlying diseases.

## Definition of hemolytic uremic syndrome

Hemolytic uremic syndrome (HUS) is the primary diagnosis for up to 4.5% of children on chronic renal replacement therapy [1–4]. It is the most common cause of acute renal failure in childhood. Following the initial description by Swiss physicians in 1955 [5], the syndrome’s nomenclature has been discussed intensively. Recently, we published our suggestions [6]. The *European Paediatric Research Study Group for HUS* [1, 6] operates a disease registry for childhood cases of HUS and encourages comprehensive investigations upon which to make valid clinicopathological and etiological correlations [1, 6–10]. In this review we focus on HUS associated with enterohemorrhagic* Escherichia coli* only (Table 1).

**Table 1:** Classification (modified from [6])

**Aetiology advanced**
1) Infection induced
(a) Shiga and verocytotoxin (Shiga-like toxin)-producing bacteria; enterohemorrhagic Escherichia coli, Shigella dysenteriae type 1, Citrobacter
(b) Streptococcus pneumoniae, neuraminidase, and T-antigen exposure
(c) other infectious agents
2) Disorders of complement regulation
(a) Genetic disorders of complement regulation
(b) Acquired disorders of complement regulation, for example anti-FH antibody
3) von Willebrand proteinase, ADAMTS13 deficiency
(a) Genetic disorders of ADAMTS13
(b) Acquired von Willebrand proteinase deficiency; autoimmune, drug induced
4) Defective cobalamine metabolism
5) Drug induced (Quinine)
**Clinical associations: etiology unknown**
1) HIV
2) Malignancy, cancer chemotherapy and ionizing radiation
3) Calcineurin inhibitors and transplantation
4) Pregnancy, HELLP syndrome and oral contraceptive pill
5) Systemic lupus erythematosus and antiphospholipid antibody syndrome
6) Glomerulopathy
7) Familial, not included in part 1
8) Unclassified

*FH* factor H; *HELLP* Hemolytic anemia, elevated liver enzymes, and low platelets; *HIV* human immunodeficiency virus; *HUS* hemolytic uremic syndrome; *TTP* thrombocytopenia

## Hemolytic uremic syndrome caused by infections

### Introductory comments

HUS caused by infectious agents is a common cause of acute renal failure in children and leads to significant morbidity and mortality during the acute phase. In addition to acute morbidity and mortality, long-term renal and extrarenal complications can occur in a substantial number of children years after the acute episode of HUS. The most common infectious agent causing HUS is enterohemorrhagic *E. coli* (EHEC). *Shigella dysenteriae* type 1 can also be associated with HUS, and as described below, HUS following infections with *Streptococcus pneumonia* can be particularly severe and has a higher acute mortality and higher long-term morbidity compared with HUS caused by EHEC [11]. D^+^ or typical HUS was linked to infection with Shiga-toxin (Stx)-producing *E. coli* in the early 1980s by Karmali et al. [7, 8, 12, 13]. Whereas the pathophysiology of HUS is beginning to be understood, more research needs to be performed to understand the precise mechanisms of cell injury in HUS so that specific therapies can be developed. Importantly, strategies to prevent EHEC infection and HUS also need to be developed and introduced to our therapeutic armamentarium.

### HUS associated with EHEC

#### Historical perspective

The original report by Gasser and coworkers described five fatal patients with hemolytic anemia, renal insufficiency, and low platelet counts [2, 7]. In these patients, it is not clear whether a gastrointestinal prodrome was evident. Almost 30 years later, Karmali et al. [7] found a cause for this disease. They showed that patients with HUS that was preceded by diarrhea contained in their stools *E. coli* strains that produced a toxin that caused irreversible damage to cultured vero cells (kidney cells from the African green monkey). Another working group demonstrated that the verocytotoxin produced by EHEC strains associated with HUS is closely related to Stx of *Shigella dysenteriae* type 1 [8]. Following this description, it was recognized that *E.-coli*-producing Stx and, as now known, other putative virulence factors, are the major causes of pediatric HUS.

#### Epidemiology

Epidemiologic studies in outbreaks of hemorrhagic colitis and D^+^ HUS have clearly shown that some patients develop hemolytic anemia and/or thrombocytopenia with little evidence of renal involvement, whereas other children develop substantial renal disease with normal platelet count and/or minimal hemolysis [7, 3, 13]. Similarly, EHEC have been isolated from children with HUS without prodromal diarrhea, making the distinction of diarrhea-positive HUS related to infection with EHEC and diarrhea-negative HUS due to other etiologies less clear.

*E. coli* O157:H7 is the serotype most commonly implicated in D^+^ HUS worldwide. However, several other non-O157:H7 EHEC serotypes are emerging [13–24]. Gerber et al. [2] described in a prospective study 394 children with HUS from Germany and Austria; 43% of the stool samples from these patients yielded serotypes others than O157:H7, including EHEC O26:H11/H^−^ (15%), sorbitol-fermenting (SF) O157:H^−^ (10%), O145:H28/H^−^ (9%), O103:H2/H^−^ (3%), and O111:H8/H^−^ (3%). In a follow-up study from the same region, the proportion of SF EHEC O157:H^−^ increased to 17% [20]. In this study, besides HUS patients who excreted EHEC-producing Stx, an additional 9% of patients shed EHEC that lost Stx genes during infection (EHEC-LST) [20].

Once a person is infected with an EHEC, the percentage of patients in whom the infection progresses to HUS depends on the infecting EHEC serotype and was reported for EHEC O157:H7 to be 15% [25]. According to our observations, the risk of HUS development is higher in patients infected with SF EHEC O157:H^−^ and lower in those infected with non-O157 EHEC (Zimmerhackl, unpublished observation). In children younger than 5 years of age, the percentage that developed hemolytic anemia or HUS was 12.9% compared with 6.8% and 8% for children aged 5–9.9 years and older than 10 years of age, respectively [26].

The use of antimotility agents was also associated with a higher risk for development of HUS [27]. A recent study demonstrated that children with hemorrhagic colitis associated with EHEC who received antibiotic therapy were more likely to progress to HUS [27]. However, a subsequent meta-analysis did not support this conclusion [27]. A study of 29 children who developed HUS found that children who received intravenous hydration and volume expansion had less severe HUS and were more likely to have nonoligoanuric renal failure [28, 29]. Environmental or genetic factors that might predispose to the progression of EHEC-associated hemorrhagic colitis to HUS are unknown. It has been suggested that alterations in the gene for factor H recently described in patients with atypical HUS may also be relevant to epidemic diarrhea-positive HUS [30, 31].

#### Pathophysiology

Stxs, which are produced by EHEC in the intestine and subsequently absorbed to the blood stream, are the major virulence factors responsible for the microvascular endothelial injury that underlies the pathophysiology of HUS [2, 31].

#### Stx structure, receptors, and transport

All members of the Stx family share a conserved A1-B5 subunit structure. They consist of a single subunit (A) of approximately 32 kDa, which is proteolytically cleaved to yield a 28-kDa peptide (A_1_) and a 4-kDa peptide (A_2_). Peptide A_1_ has enzymatic activity, and peptide A_2_ connects the A subunit to the pentamer of five identical B subunits. The B subunit of Stx may contain several binding sites for its glycosphingolipid receptor Gb3Cer, which is presented on endothelial cells [2, 33]. After binding to Gb3Cer at the cell surface, Stx is endocytosed and retrogradely transported to the Golgi apparatus and the endoplasmic reticulum. It is then translocated to the cytosol where it inactivates ribosomes, thereby causing cell death [32]. The ribosome-inactivating subunit is the A1 chain that possesses ribosomal ribonucleic acid (rRNA) N-glycosidase activity. In some cells, Stx is endocytosed mainly by clathrin-dependent endocytosis [32], although other mechanisms also exist [34]. It has been shown that the fatty acid of Gb3 is important for efficient transport of Stx to Golgi apparatus. Therefore, the composition of Gb3 may play a role in the endocytic pathway used. A raft localization of StxB was recently found to be required for efficient retrograde transport [35]. Toxicity induced by Stx is influenced by cytokine release. In particular, tumor necrosis factor alpha (TNF-α) is able to increase Gb3 receptor density and thus increase toxicity to endothelial cells [36].

#### Heterogeneity of Stx

Sequence analysis of *stx* genes and toxin neutralization assays in EHEC strains isolated from patients have shown the existence of two major Stx families, Stx1 and Stx2, each of which contains the major Stx type and an increasing number of variants. The Stx1 family at the present time consists of Stx1, Stx1c [37], and Stx1d [38]. The more heterogeneous Stx2 family comprises variants designated Stx2c [39], Stx2c2 [40], Stx2d [41], Stx2d_activatable_ [41, 42], Stx2e [43], and Stx2f [44]. Stx2d_activatable_ differs from all known Stx types in that it can be activated in its biological activity by elastase [42, 45], a constituent of the intestinal mucus that cleaves the last two C-terminal amino acids of the A_2_ peptide of the Stx A subunit [46]. A single strain can possess one or more different *stx* genes [18, 19, 42, 47]. Stx2 genes can be duplicated as it was recently demonstrated [48]. Whereas *stx*_1_, *stx*_2_, *stx*_2c_, and *stx*_2dact_ genes are usually expressed [18–20, 23, 42, 48], discordance between the *stx* genotype and Stx expression in strains harboring *stx*_2d_ or *stx*_2e_ has been demonstrated [49]. The control of expression in strains producing Stx and those not producing Stx appears to be at the level of transcription [49]. Thus, the detection of Stx using antibodies and cell culture assays might fail in isolates that are poor secretors of Stx.

#### Association between the Stx type and HUS

The clinical outcome of an infection involving Shiga-toxin-producing *E. coli* (STEC) depends mainly on the type of Stx produced by the infecting strain and the possession of non-Stx virulence factors. Subtyping of *stx* genes in large collections of EHEC strains isolated from clinically well-defined subjects demonstrated that EHEC producing Stx2, Stx2c, or Stx2d_activatable_ were responsible for severe disease such as hemorrhagic colitis and HUS [18, 37, 42, 50]. *E. coli* strains producing Stx1c, Stx1d, Stx2d, and Stx2e were associated with uncomplicated diarrhea and asymptomatic infections [38, 39, 43, 47, 51]. Whereas strains producing Stx2 and/or Stx2c usually possess the intimin-encoding *eae* gene, which is associated with high virulence [47], strains producing Stx2d_activatable_ are *eae* negative [42]. It can be concluded therefore that highly pathogenic EHEC organisms are usually *eae* positive but that *eae* negative EHEC exist, which can also cause severe disease in humans. Presumably, production of a highly toxic agent activated by mucus in vivo may compensate for the absence of intimin, the molecule that mediates the intestinal adhesion of *eae*-positive EHEC and thus promotes the efficient transport of Stx from the intestine into the bloodstream. Because the risk of HUS following infection with *E. coli* strains harboring different *stx* alleles varies, there is need for rapid and comprehensive subtyping of *stx* genes in STEC isolates at an early stage of the illness [42, 47]. This information will aid risk analysis and prediction of clinical outcome of the infection.

#### Loss of Stx during infection

Genes encoding Stx are encoded in the genomes of lambdoid prophages, which are also called Stx-converting bacteriophages, or Stx-phages [52–54]. *Stx* is under the regulation and control of phage genes, and replication of the prophages will result in an increase in *stx* gene dosage [52, 54]. *Stx* genes are induced in EHEC strains at a very low level spontaneously. A dramatic replication of the phage genomes and thus multiplication of *stx* genes occurs after exposure of sublethal doses of ultraviolet (UV) light, mitomycin C, and various antibiotics [55, 56] and other stimuli such as H_2_O_2_ released from neutrophils [57]. Prophage induction also leads to phage-mediated lysis of the *E*. *coli* cell envelope, which seems to be important for release of Stx, as no specific Stx transporter proteins have been identified [52–54]. Although phage-mediated lysis and Stx release seem to represent the same event [53, 54], other phage-independent mechanisms, such as lysis by particular colicins, may contribute to Stx release in vivo [58]. Following induction, Stx phages can infect other bacteria in vivo and in vitro if these carry a phage receptor and a free integration site for the phage [52, 59–61]. Therefore, Stx phages are critical for production of Stx, for Stx release and for the dissemination of *stx* genes.

Phages also participate in the loss of *stx* genes. We recently demonstrated that several EHEC serotypes lost Stx-converting phages during infection and thus the capability to produce Stx [22, 60, 61]. These events involve a change in pathotype of the infecting organism and thus might contribute to an altered virulence during the course of infection and pose a diagnostic challenge [20]. This is due to the fact that procedures that rely on the detection of Stx or *stx* genes are routinely used to screen for EHEC in clinical laboratories and that mostly only a single stool sample, collected late in the illness, is available for investigation [20].

#### Histology

**Fig. 1:**
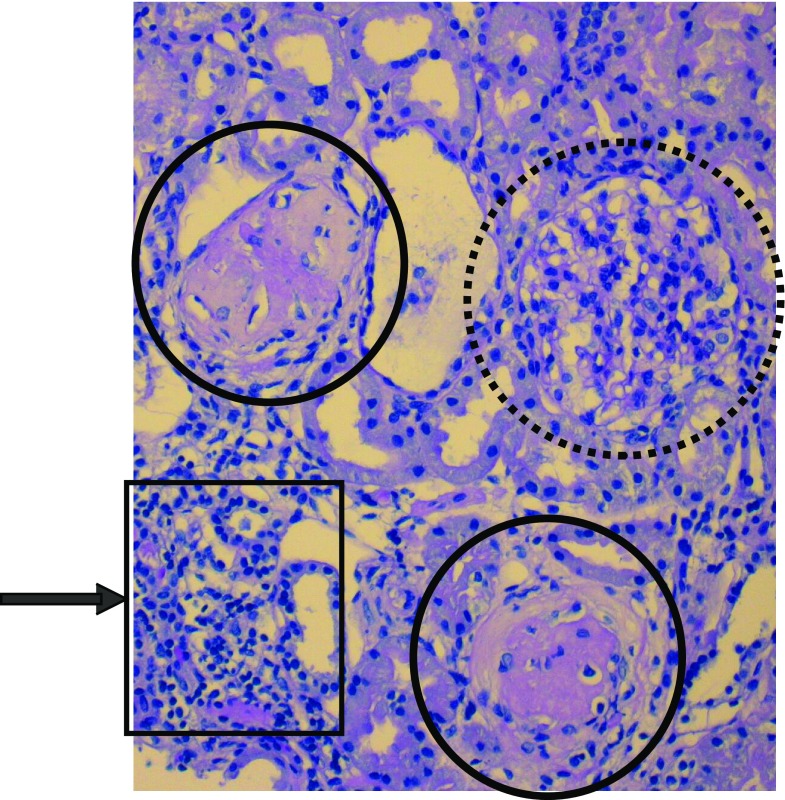
Hematoxylin and eosin staining in hemolytic uremic syndrome (HUS). Note that two glomeruli are completely sclerosed (*solid line*). Mesangial expansion with beginning sclerosis in the third glomerulus (*broken line*). Focal inflammation in the tubular system indicating involution of renal parenchyma (*arrow*). Courtesy of Prof. Dr. Consolato Sergi

In the histology, the arteriolar afferentes—and more rarely, the arteriolar efferentes—show these variances: swelling of the endothelial cell, subendothelial deposits of fibrinoids substances, and thrombosis of the arterioles. In the glomerulus, swelling of capillary endothelial cells and capillary dilatation are found. Further deposits of fibrin in the capillary, including thrombosis and hyalinosis, are described (Fig. 1). Tubular damage, focal or segmental, with necrosis and atrophy is of significance for long-term outcome. Changes of the interstitium are described, but there value is unclear. The differences in the arterioles and glomeruli are more significant than the tubulointerstitial variances [62–66]. From experimental studies with Stx, it can be concluded that almost all cell types are involved. Damage to endothelial cells, mesangial cells, tubular cells, and also the podocyte are known. The role of the individual compartment is unclear. However, Stx alone is not the only toxic factor. Lipopolysaccharide can damage renal cells as well. The ability to injure human microvascular endothelial cells has been demonstrated for EHEC hemolysin [67], cytolethal-distending toxin [68, 69] found in EHEC O157:H7 [69], and non-O157 EHEC causing HUS [70]. Moreover, an enzymatically active form of serine protease EspP, termed EspPα, that cleaves factor V, is frequently found in the EHEC serotypes mostly associated with HUS [71]. In addition, the inflammatory response contributes to kidney damage [72]. Thus, it seems, that the basic disease is an endothelial lesion with secondary thrombosis [63, 64, 72–74].

#### Clinical manifestations of D^+^ HUS

**Fig. 2:**
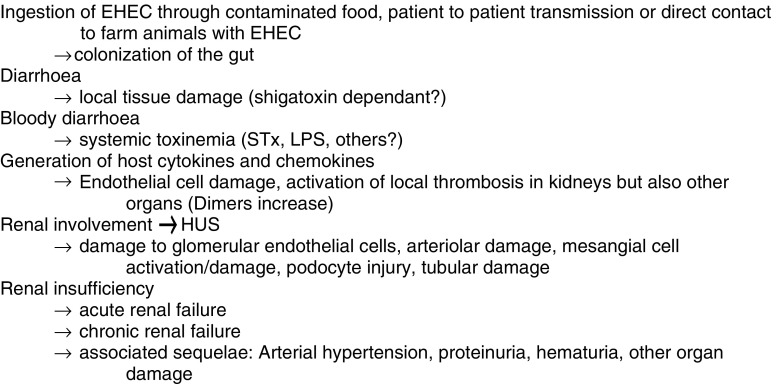
Development of Shiga-toxin-associated hemolytic uremic syndrome (used with permission from [72])

**Fig. 3:**
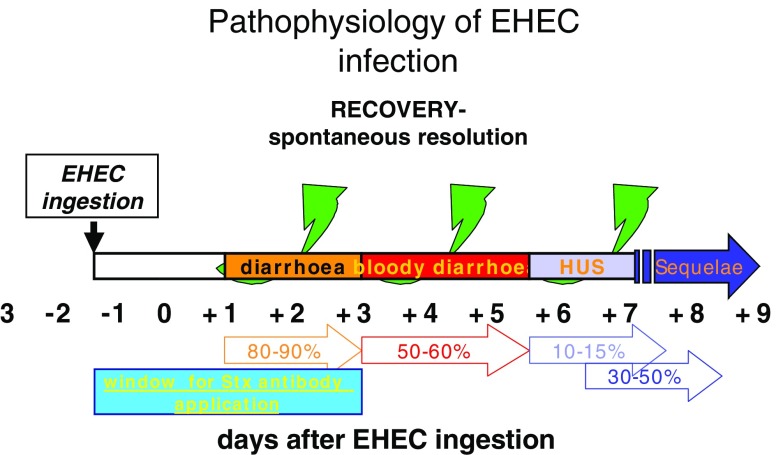
Pathophysiology of enterohemorrhagic *Escherichia coli* infection. Prodromal phase usually 3 days. Window for Shiga toxin antibody treatment is from day 0 to day 3 after onset of diarrhea. Sequelae in percent of patients. Adapted and modified from [24]

After an incubation period of 3–8 days, patients develop watery diarrhea followed by bloody diarrhea accompanied with abdominal cramps in the majority of cases (Figs. 2 and 3). About 50% of these patients develop nausea and vomiting. Only 30% have fever. Use of antimotility agents and antibiotics [75, 76], bloody diarrhea, fever, vomiting, elevated serum leukocyte count, extremes of age (<5 years) and female gender have been associated with an increased risk for HUS following EHEC infection. These symptoms are followed by the typical hematological and nephrological alterations. Patients suffer from hemolytic anemia, thrombocytopenia, and renal failure. The clinical picture is marked by increasing anemic pallor and oliguria or anuria. Furthermore, edema, arterial hypertension, proteinuria, and hematuria can occur. Other characteristics are fragmented erythrocytes, acute decay of hemoglobin, massive increase of lactate dehydrogenase (LDH), low or undetectable levels of haptoglobin, and thrombocytopenia.

Depending on the renal damage, high renal retention values and decrease of creatinine clearance can be found [77]. Whereas the kidney and gastrointestinal tract are the organs most commonly affected in HUS, evidence of central nervous system, pancreatic, skeletal, and myocardial involvement may also be present [65, 66, 78]. Gastrointestinal involvement with severe colitis can result in transmural necrosis with perforation and/or the later development of colonic stricture [66, 78]. Elevation of pancreatic enzymes is common, and edema of the pancreas, indicative of pancreatitis, can be detected by ultrasound or computed tomography (CT) scan [79]. Central nervous system (CNS) involvement in typical HUS is common and frequently presents as lethargy, irritability, and seizures, and in more severe cases, CNS disease presents as paresis, coma, and cerebral edema. Skeletal muscle involvement manifested as rhabdomyolysis occurs in rare cases, and fortunately, myocardial involvement is rare as well [80, 81]. When myocardial involvement occurs, elevated troponin I level may reflect the degree of myocardial ischemia [81]. HUS mortality is reported to be between 3% and 5%, and death due to HUS is nearly always associated with severe extrarenal disease, including severe CNS disease [66, 76, 82].

#### Antibiotic treatment and potential preventive agents

There is a long history of the discussion of antibiotic treatment for EHEC-induced diarrhea. Ever since in vitro studies demonstrated that EHEC produces more toxins when stimulated by nonlethal concentrations of antibiotics, this issue has been under controversial discussion.

During the large EHEC outbreak in Japan in 1996, it was suggested that treatment with Fosfomycin on day 2 after disease onset reduced the risk of developing HUS. That study has several drawbacks. In particular almost all patients were treated, an unusual recommendation in other parts of the world. In addition, Fosfomycin is rarely used for this indication at all outside of Japan. Furthermore, recent epidemiological studies from St. Louis conducted by the US Centers for Disease Control indicated that antibiotic therapy for EHEC enteritis resulted in a significantly higher risk of developing HUS [83]. This adverse outcome may reflect the effect of specific antimicrobial agents on phage induction and subsequent Stx gene expression and transcription or increased Stx release after induced bacteria lysis [17, 25–27].

Some studies demonstrated a harmful effect of antibiotic therapy in hemorrhagic colitis. Children with hemorrhagic colitis associated with EHEC who received antibiotic therapy were more likely to develop HUS compared with children who did not receive antibiotic therapy [26, 27, 84]. Other studies have not demonstrated such an association, and a recent meta analysis concluded that administration of antibiotics in people infected with EHEC was not associated with the development of HUS. In in vitro studies, it has been shown that some antibiotics promote production and release of Stx from *E. coli*. Currently, there is no consensus on the use of antibiotic therapy in children with hemorrhagic colitis or HUS; however, antibiotics are not usually prescribed in children with HUS until there are specific indications for antibiotic therapy. In conclusion, during the diarrheal phase, antibiotic treatment should be avoided, as beneficial effects regarding initiation of HUS cannot be deduced from recent studies [25, 84, 85]. Antibiotic treatment after HUS onset has not been shown to be of negative influence for long-term outcome (personal observation, LBZ).

#### Other preventive strategies

A diatomaceous silicon diamide compound linked to an oligosaccharide chain (Synsorb® Pk) was developed and shown to avidly bind and neutralize Stx. A clinical trial was recently completed to determine whether oral administration of Synsorb® Pk can decrease the rate of progression of hemorrhagic colitis to HUS or whether it can decrease the need for dialysis or extrarenal complications in children who have developed HUS. Unfortunately, the Synsorb® Pk was not found to be beneficial in preventing extrarenal complications or decreasing the duration of dialysis in children with new-onset HUS. Starfish is a new compound shown to bind to Stx 1,000 times more efficiently than Synsorb® Pk. Starfish is a pentameter that binds Stx and has the potential to be administered intravenously. Starfish has been shown to protect mice against a lethal dose of Stx1 but not Stx2, whereas a modified version of Starfish, called Daisy, protected mice against lethal doses of Stx1 and Stx2 [86, 87].

#### Stx antibodies

Very interesting results have been obtained from studies that demonstrated that monoclonal antibodies specific for the A subunit of Stx2 prevented lethal complications in mice when administered after diarrhea onset. The authors suggested that treatment of children with this antibody after the onset of bloody diarrhea may be protective against HUS development. Other very interesting recent studies demonstrated that vaccination with a plant-based oral vaccine protected mice against a lethal systemic intoxication with Stx2 [88].

#### Dialysis

The majority of children with HUS develop some degree of renal insufficiency. Approximately two thirds of children with HUS will require dialysis therapy, and about one third will have milder renal involvement without the need for dialysis therapy [2]. Peritoneal dialysis and hemodialysis modes have been used in the past. In most centers, peritoneal dialysis is the preferential choice. However, there is no priority to one or the other. We recommend that hemodialysis may be started if atypical HUS is suggestive. This is particularly true in older children and those without clear diarrhea. In younger children, most centers prefer peritoneal dialysis. It has been argued that PD may have a higher risk of peritonitis in patients with bloody diarrhea. However, this has not yet been reported [75].

Thus, HUS management encompasses the usual management of children with acute renal failure with additional management issues specific to HUS. General management of acute renal failure includes appropriate fluid and electrolyte management, antihypertensive therapy if the child demonstrates hypertension, and initiation of renal replacement therapy when appropriate [76]. Specific management issues in HUS include managing the hematological complication of HUS, monitoring for extrarenal involvement in HUS, avoiding antidiarrheal drugs, and possibly avoiding antibiotic therapy. Managing hematological complications of HUS, including hemolytic anemia and thrombocytopenia, should involve frequent laboratory studies to include a hemoglobin and hematocrit determination on a frequent schedule, as children may undergo rapid hemolysis.

In addition, jaundice may develop due to the hemolytic process and is characterized by an increase in indirect bilirubin. Transfusion with packed red blood cells is needed when the hemoglobin is falling rapidly and/or when the hemoglobin reaches 6–7 mg/dl. Children should be transfused with packed red blood cells over a 2- to 4-h interval with diuretic therapy, as indicated if the child has evidence of volume overload. Careful monitoring of blood pressure, urine output, and respiratory status are important to assure that the child does not develop pulmonary edema.

Thrombocytopenia can be profound, but platelet transfusions are usually limited to the need for a surgical procedure or in active bleeding. The rationale for limited platelet transfusions is that they can contribute to the development of microthrombi and promote tissue ischemia, with an aggravation of HUS symptoms, in particular, neurological deterioration. Since microthrombi form during the course of HUS in multiple organs, including the kidney, central nervous system, colon, pancreas, skeletal muscle, myocardium, and other organs, accelerated deposition of microthrombi may occur following platelet transfusions and promote tissue injury. Intravascular volume needs to be considered when a transfusion is indicated, as many children with acute renal failure due to HUS are oliguric and at risk for fluid overload and pulmonary edema.

The kidney and gastrointestinal tract are the organs most commonly affected in HUS, but other organs are also affected in a substantial number of children. CNS involvement may be manifested as irritability, seizures, and/or coma. In some patients, pancreatitis with or without glucose intolerance will develop during the acute phase of the disease, whereas skeletal and myocardial involvement may also be present. It is very important to evaluate the presence and extent of extrarenal involvement, as these complications of HUS are what contribute to the mortality of HUS. In children with HUS, physical examination and appropriate laboratory studies are needed to monitor for the development of extrarenal manifestations. The neurological examination screens for CNS involvement, and radiographic imaging is needed in symptomatic patients, including those with combativeness, irritability, seizures, and decreased level of consciousness. In addition to monitoring the level of renal function, hemoglobin, hemolytic parameters (LDH, haptoglobin), hematocrit, and platelet count, as described above, amylase, lipase, glucose, and liver function studies should be performed during the acute phase of the disease.

In children with hemorrhagic colitis due EHEC infection, the use of antimotility agents has been associated with a greater risk for developing HUS. Thus, antidiarrheal agents are usually avoided, as it is thought that this contributes to retention of Stx within the colon, which could enhance absorption of the toxin [25, 72, 75, 76, 84].

#### Plasma therapy

While clearly indicated in some children with atypical HUS, therapy with plasma infusion [89] and or plasma exchange [90] has proven to be beneficial in Stx-associated HUS [89, 90]. In situations where the child is in jeopardy, in particular with neurological symptoms, plasma exchange is used. Nevertheless, it should be stressed that there is no evidence that such procedure is beneficial to the patient (personal observation, LBZ).

#### Prognosis: long-term outcome

HUS prognosis depends on several contributing factors. In general “classic” HUS induced by gastrointestinal bacteria (EHEC) has an overall better outcome than does atypical HUS. HUS caused by pneumococci is probably hampered by more severe active and chronic sequelae. Totally different is the prognosis in patients with atypical and, in particular, recurrent disease. In a small study in Europe, the outcome after 1 year was significantly worse regarding renal function and increased arterial blood pressure. The group with EHEC-associated HUS had a normal glomerular filtration rate (GFR) on average and normal blood pressure, whereas patients with recurrent disease had a GFR on average in renal insufficiency grade 3. The same was true for arterial blood pressure, which was significantly elevated in two thirds of patients. The situation after transplantation seems to be even more difficult. This issue will be addressed later [10, 88].

As dialysis techniques are available for all age and weight groups, the prognosis of renal failure in HUS has improved significantly. Furthermore, improvement in handling these children by pediatric nephrologists has resulted in better survival. Some children never recover renal function and require long-term renal replacement therapy, whereas those who recover are at risk for late development of renal disease.

HUS is not a kidney disease. In many children, extrarenal symptoms occur. Neurological symptoms such as seizures are present in a quarter of patients. Therefore, some children have residual extrarenal problems, including neurological defects, insulin-dependent diabetes mellitus (IDDM), pancreatic insufficiency, and/or gastrointestinal complications. Thus, HUS is a disease with substantial acute and chronic mortality and multisystem morbidity [82, 91, 92].

Several studies have demonstrated that children who have recovered from the acute episode of HUS are at risk for long-term complications, including hypertension, renal insufficiency, end-stage renal failure, and IDDM. One study found that 39% of 61 children with a history of HUS demonstrated late complications, including hypertension, proteinuria, and renal insufficiency during a mean of 9.6 years after the acute episode. The duration of oligoanuria was found to be the best predictor of late complications [75, 76, 82]. Other studies have demonstrated that histological findings of focal and segmental sclerosis and hyalinosis are observed several years following HUS. In this study, only a quarter of the children had normal renal function during long-term follow-up [85]. Kidney biopsies performed in children with a history of HUS and residual proteinuria demonstrated that the majority of these children had global and segmental sclerosis with interstitial fibrosis, suggesting that they were at risk for later development of renal insufficiency. In addition, ambulatory blood pressure monitoring was abnormal in several children with a history of HUS and normal casual blood pressure. A meta-analysis demonstrated that death or end-stage renal disease (ESRD) occurred in 12% of children with diarrhea-associated HUS, and 25% of survivors demonstrated long-term renal sequelae [66, 85]. The role of arterial hypertension is obvious. The likely increased glomerular pressure associated with hyperfiltration in the regenerative phase of HUS might render inhibition of the renin-angiotensin system beneficial. Although no evidence-based information or a study of this subgroup is available, angiotensin-converting enzyme (ACE) inhibitors or angiotensin receptor blockers (ARB) are preferentially used by pediatric nephrologists. This is the best treatment available based on the pathophysiological information.

Children with HUS who were discharged without neurological injury did not have an increased risk for subclinical problems with learning behavior or attention, whereas some children who had major neurological symptoms had evidence of subtle neurological sequelae, including clumsiness, poor fine-motor coordination, hyperactivity, and distractibility. Long-term gastrointestinal complications, such as colonic stricture and bilirubin gallstones, can develop following apparent recovery of HUS. Permanent or transient IDDM occurs in a small percentage of children with HUS, and children who have transient IDDM are at risk for later return of IDDM. Interestingly, these children do not have anti-islet cell antibodies, and the pathogenesis of their IDDM is not related to immunologic injury but rather to decreased beta cell function [66, 79].

#### Renal transplantation

Renal transplantation in “classic” HUS is rare. Recent reviews support the notion that in classic HUS, a recurrence of HUS is the absolute exception. Therefore, in patients with Stx-associated HUS, transplantation can be performed without increased risk for transplant failure [10, 93].

#### Accepted treatment of patients with diarrhea-associated HUS

Despite the increased understanding of the pathophysiology of EHEC-associated HUS, treatment modalities have not changed over recent decades. It has been demonstrated that almost all pharmacological interventions are harmful or at least do not improve acute and long-term outcome. However, it is of particular importance to identify patients with a complement disorder. These patients will very likely benefit from plasma therapy.

#### Experimental strategies in patients with D^+^ HUS

There are several therapeutic strategies on the list. They include immunization against parts of the Stx molecule, humanized monoclonal antibodies against Stx, as mentioned above, and inhibition of TNF-α, cytokines, and complement factors. Furthermore, the idea of binding the released Stx in the gut via amorphic compounds is still under investigation. No patient is currently under clinical testing. Thus, our armamentarium to cure HUS is still limited. However, the hope that a better understanding of this diverse disease group will produce better therapies is the driving force for intensified research.

#### Diagnostic proposals in patients with HUS

Diagnostic workup of patients with HUS has been proposed by a group of European nephrologists organized in the European Study Group on Hemolytic Uremic Syndrome and Related Disorders. According to the suggestions of this group, the cause of HUS should be determined. It should be kept in mind that infectious causes may be linked to other underlying diseases. Thus, even when EHEC association is likely, the other causes should be considered. A detailed description with more hints also for atypical HUS is listed under the home page of the European Society for Paediatric Nephrology:

https://doi.org/espn.cardiff.ac.uk/hus_guideline_2005.pdf

#### Future

##### Anti-Shiga toxin antibodies

Anti-Stx antibodies have been shown to prevent HUS in animals. There are several authorized products on the market. In December 2005, the US Food and Drug Administration (FDA) approved orphan drug status for two chimeric anti-Stx antibodies (caStx1 and caStx2, made by Caprion© Pharmaceuticals, Inc) in the treatment of STEC infections. The antibodies are intended to neutralize circulating Stx1 and Stx2, thereby treating the disease and preventing serious complications such as gastrointestinal disease, bloody diarrhea, destruction of red blood cells and platelets, and HUS. The product is being evaluated for preventing HUS in a dose-escalating, phase 1, US clinical trial of STEC-infected pediatric patients. It was also recently designated as an orphan drug for this indication by the European Medicines Evaluation Agency. The major drawback is that the window for application is very small. In particular, the diagnosis of EHEC has to be made within 5 days after inoculation or within 2 days after initiation of diarrhea, a challenge that cannot be achieved routinely with current techniques. However, new diagnostic tools are under development (see also Fig. 3 for window of Stx-antibody therapy).

Immunization protocols are under investigation. None is ready for use in the near future. Complement inhibition can prevent thrombosis in paroxysmal nocturnal hemoglobinuria (PNH), a disease with a membrane-bound complement-inhibitor defect [92]. Under the assumption that Stx-associated HUS may cause damage to renal cells via local complement activation, inhibition of complement activation with a C5 antibody maybe beneficial [94].

In conclusion, an improved understanding of the mechanisms of Stx infections and the pathophysiology of cell injury in HUS will lead to new therapeutic strategies for children with HUS to prevent the acute mortality and the long-term morbidity of HUS in the near future. Therefore, research is necessary, and international preventive strategies are mandatory.

https://doi.org/www.hemolytic-uremic-syndrome.org

https://doi.org/www.hus-online.at

## Multiple-choice questions

(Answers appear following the reference list)

1. How many patients with Stx-producing *E. coli* infections develop HUS?
  A. 100%
  B. <1%
  C. 5–15%, and it depends on the age of patients
  D. 50–60%
  E. All patients with Stx1-producing* E. coli*
2. Which of the following is associated with a poor HUS prognosis? (more than one answer is possible)
  A. Antibiotic therapies
  B. CNS involvement
  C. Patients <5 years
  D. Female patients
3. Stx is proposed to be the noxious agent in HUS. There are several types of Stx. Which one is not strongly associated with HUS?
  A. Stx2
  B. Stx2d_activatable_
  C. Stx2c
  D. Stx1
4. EHEC is defined as having at least three virulence factors. Which one does not belong to them?
  A. Intimin encoded by *eae* gene
  B. EHEC hemolysin
  C. Staphylolysin
  D. Stx
  E. Verocytotoxin
5. For platelet transfusion in HUS, the following statements are correct (one right answer)
  A. Transfusion necessary in all patients with platelet counts <50,000
  B. Transfusion necessary only when signs of bleeding
  C. Transfusion necessary in all patients with need for surgery
  D. Transfusion necessary if platelet counts <30,000
6. For dialysis in HUS patients, which of the following statements is correct
  A. Hemodialysis is better than PD
  B. PD is better than HD
  C. Dialysis is necessary in all patients to remove the toxin
  D. HD is the preferred treatment when plasma therapy is considered
  E. Dialysis is necessary if blood urea nitrogen is >50
7. HUS can be prevented by oral application of silica gel, which binds Stx.
  A. True
  B. False
8. Which of the following statements is correct?
  A. HUS has the same incidence around the world
  B. HUS is more common in Argentina than the USA
  C. HUS is mainly caused by adenovirus
  D. HUS is the disease of the adolescent child
  E. HUS can be prevented by immunization
9. Hemolytic anemia in HUS is Coombs positive. This statement is
  A. True
  B. False
10. Patients with HUS usually benefit from (one correct answer)
  A. Plasma infusion
  B. Plasma exchange
  C. Glucose infusion
  D. Appropriate fluid balance
  E. Steroids

### Answers

1 C, 2 A, B, 3 D, 4 C, 5 B, 6 D, 7 B, 8 B, 9 B, 10 D

## Abbreviations

HUS: hemolytic uremic syndrome
VTEC: verocytotoxin-producing *Escherichia coli*
Stx: Shiga toxin
EHEC: enterohemorrhagic *Escherichia coli*
FH: factor H
FI: factor I
CD46: membrane-bound cofactor of the complement system (MCP)
